# Open Access of COVID-19-related publications in the first quarter of 2020: a preliminary study based in PubMed

**DOI:** 10.12688/f1000research.24136.2

**Published:** 2020-08-12

**Authors:** Olatz Arrizabalaga, David Otaegui, Itziar Vergara, Julio Arrizabalaga, Eva Méndez

**Affiliations:** 1 Innovation Group, Biodonostia Health Research Institute, San Sebastian, 20014, Spain; 2 Multiple Sclerosis Group, Biodonostia Health Research Institute, San Sebastian, 20014, Spain; 3 Group of Research in Primary Care, Biodonostia Health Research Institute, San Sebastian, 20014, Spain; 4 Library and Information Science Department, Universidad Carlos III de Madrid, Madrid, 28903, Spain

**Keywords:** Open Access, Publishing, Pandemic, COVID-19, Scholarly communication, PubMed, OA analysis.

## Abstract

**Background:** The COVID-19 outbreak has made funders, researchers and publishers agree to have research publications, as well as other research outputs, such as data, become openly available. In this extraordinary research context of the SARS CoV-2 pandemic, publishers are announcing that their coronavirus-related articles will be made immediately accessible in appropriate open repositories, like PubMed Central (PMC), agreeing upon funders’ and researchers’ instigation.

**Methods:** This work uses Unpaywall, OpenRefine and PubMed to analyse the level of openness of the papers about COVID-19, published during the first quarter of 2020. It also analyses Open Access (OA) articles published about previous coronavirus (SARS CoV-1 and MERS CoV) as a means of comparison.

**Results:** A total of 5,611 COVID-19-related articles were analysed from PubMed. This is a much higher amount for a period of 4 months compared to those found for SARS CoV-1 and MERS during the first year of their first outbreaks (337 and 125 articles, respectively).  Regarding the levels of openness, 97.4% of the SARS CoV-2 papers are freely available; similar rates were found for the other coronaviruses. Deeper analysis showed that (i) 68.3% of articles belong to an undefined Bronze category; (ii) 72.1% of all OA papers don’t carry a specific license and in all cases where there is, half of them do not meet Open Access standards; (iii)  there is a large proportion that present a copy in a repository, in most cases in PMC, where this trend is also observed. These patterns were found to be repeated in most frequent publishers: Elsevier, Springer and Wiley.

**Conclusions:** Our results suggest that, although scientific production is much higher than during previous epidemics and is open, there is a caveat to this opening, characterized by the absence of fundamental elements and values ​​on which Open Science is based, such as licensing.

## Introduction

In the first four months (January–April), due to the COVID-19 pandemic, funders ^1, 2^, researchers and publishers (such as Springer or Wiley) seem to agree upon making research outcomes related to the SARS CoV-2 pandemic openly available, including research papers (from preprints - MedRxiv and bioRxiv - to different mechanisms for waiving Article Processing Charges ( APCs) or new specific Open Research platforms, as Elsevier or The Lancet). However, traditional practices for scholarly publishing and regular practices to access scientific content might not be mature enough for this massive open endeavour.

Throughout history, research and innovation have been key in the transformation of our society. It has been observed that, in addition to a direct economic benefit, only those societies with a certain level of scientific culture have the capacity to face new risks and participate in new ethical dilemmas, like the ones that we are currently facing. The more scientifically educated societies are, the freer they become, since answers to big social challenges arise from this interaction ^3^. Open Access (OA)/Open Science has been promoted over the last few decades by different stakeholders of the scientific system to make publications openly accessible, and more recently, also data and other research outcomes, in order to make them FAIR (Findable, Accessible, Interoperable and Reusable). All these initiatives aim to boost a democratic scientific advance in which scientists but also citizens are involved.

In the current situation of a global pandemic, OA becomes urgent. The emergence of the virus that causes the disease known as COVID-19 first reported by the Chinese authorities in late December 2019, has resulted in an unprecedented level of collaboration among researchers around the world ^4– 6^. A health crisis, such as the SARS CoV-2 pandemic, requires special effort and collaboration within the scientific community in order to generate and disseminate new results, while trying to avoid duplication of efforts globally.

In this unique context of the pandemic, publishers are announcing massive OA changes, primarily by making their coronavirus-related articles freely available through databases, such as PubMed Central (PMC), together with other public repositories. SPARC Europe stated that overnight COVID-19 heightens the need for Open Science, and we cannot agree more. But we wonder if this openness might be enough in such a demanding and urgent episode for Science, and coincidently we wonder if the scientific community is ready to share and consume openly such information. This work aims to make an initial analysis of scientific production concerning COVID-19 and its level of openness as a first step to assess the current research publication model and the unpredicted outcome of openness of research in this global health emergency. Thus, this paper analysed all scientific content openly available from PubMed database.

In addition, results were compared with the scientific production about other epidemics such as SARS CoV-2 and MERS CoV, due on the one hand, to the similarity in their epidemiological burden based mainly on their respiratory transmission (unlike other epidemics such as Ebola or Zika), and on the other by the alarm generated during the first outbreaks.

## Methods

### Data source

In order to analyse publications concerning COVID-19 and their level of openness, we have chosen PubMed instead of other multidisciplinary bibliographic databases, like Web of Science (WoS) or Scopus for three main reasons:

a) PubMed, a database developed by the National Center for Biotechnology Information ( NCBI) at the National Library of Medicine ( NLM) in the USA, is one of the most used databases to find biomedical scientific content. This database gathers over 14 million bibliographic citations and it provides access to MEDLINE articles and PubMed Central (PMC), an extensive digital repository created in 2000 for biomedical and life sciences Open Access publications.
b) Unlike many other research databases, such as WoS, PubMed also includes articles that are “in process”; this means a status prior to being indexed with MeSH terms, and articles submitted by publishers as pre-prints (i.e. articles that haven't gone through peer review) ^7^. This aspect is crucial for this study since, at this moment, scientific papers are being published very fast and may not have yet undergone peer review ^8^.
c) In response to the COVID-19 Public Health Emergency, many publishers have promised to make their coronavirus-related articles freely available in PMC and other public repositories. Thus, being PMC part of PubMed ^9^, it is appropriate to use this database in order to further inquire into the content of PMC.

Finally, after an overview carried out by the authors of this paper prior to this study in other databases, it was concluded that PubMed, unlike WoS, was also the one with more up-to-date Open Access information during the analysed period of this COVID-19 outbreak. So, if an article was OA or free to read, it could be reached through PubMed.

### Search terms

Since during the global pandemic period, the scientific community is posting articles that are freely accessible through the NCBI, data were collected from the PubMed database in order to analyse every COVID-19-related scientific paper that is currently published (including PMC) ^9^. In an attempt to evaluate the most accurate list of publications, we exported all results obtained from the suggested search queries offered by NLM ( NCBI webpage), as follows: “2019-nCoV OR 2019nCoV OR COVID-19 OR SARS-CoV-2 OR (wuhan AND coronavirus)”. Only articles published from January 1 ^st^ to April 23 ^rd^ of 2020 were considered. No exclusions were made in the type of article (journal article, books, reviews, clinical trial or meta-analysis) or in the language, choosing in each case every article offered by PubMed. No preprints were found in the returned results.

In line with the objective of analysing published papers during other emergency circumstances, similar search procedures were applied to the SARS CoV-1 pandemic (query: “SARS CoV” OR “Severe Acute Respiratory Syndrome Coronavirus”; period searched: from 2003 to 2006) and MERS CoV epidemic (query: “MERS CoV” OR “Middle East Respiratory Syndrome Coronavirus”; period searched: from 2013 to 2016).

In order to determine the effect that this health emergency is having on the availability of the scientific production, we decided to compare it with the availability in a normalized situation, for which we performed the same analysis using two chronic diseases: low grade glioma (query: “low grade glioma”) and peptic ulcer (query: “peptic ulcer”), which, as seen by our search, have stable publication patterns for the last three years (from 2017 to 2019).

### Data analysis

Obtained results, without exclusion, were exported and uploaded to OpenRefine, a free open source tool that helps exploration of large data sets, and has the capability to link and extend these data sets with different web services. In this study, OpenRefine was used to manage data but also as the key element in order to link our PubMed data set with Unpaywall through its application programming interface (API), the selected tool for analysing the OA content of all these data. Unpaywall (previously known as oaDOI) is a database introduced in 2016 as a service to check OA availability of journal articles identified by their Digital Object Identifier (DOI) ^10^. Unpaywall is currently used more than 50,000 times a day and is maintained by Our Research, a non-profit company previously called Impactstory ^11^. It offers access to the OA status of scientific journals, through an open API. Unpaywall also shows license information and variable version availability from different repositories ^10, 11^. In this study, Unpaywall data was collected via API in OpenRefine at the moment of the study (April 23 ^rd^), but also at the review process of the work (in mid-July) after an update carried out in Unpaywall team and communicated to the authors of this paper. So both, data and methods are reviewed and updated in this version of the paper. This underlines the importance of ‘real-time science’ measurement, in a ‘real-time research’ publication process, like the one reflected in this paper.

Based on Unpaywall categorisation ^12, 13^, four types of OA are considered: Gold, journal which publishes all its papers in Open Access without taking into account its business model. Here are included journals indexed by the Directory of Open Access Journals ( DOAJ) but also other 100% OA journals precisely added by Unpaywall; Hybrid, subscription-based (non-OA) journals including some OA articles upon a fee, charged to the authors; Green, self-archived versions of a paper in a repository. It could be toll-access on the publisher page, with a free copy in an OA repository after an embargo period, or it could also be a Gold or Hybrid paper that the author has self-archived; and Bronze, articles freely available on websites hosted by their publisher, either immediately or following an embargo, but are not formally licensed for reuse ^12^. Unpaywall also provides information about Creative Commons (CC) licensing of each document (commonly Gold OA or hybrid journals). Copyright licenses, released by Creative Commons, are variable and range from more open - and therefore more reusable (CC0, Public Domain (PD), CC-BY or CC-BY-SA) to more restrictive ones (CC-BY-ND, CC-BY-NC, CC-BY-NC-ND or CC-BY-NC-SA) ^14^. In addition to these types of licenses, Unpaywall also returns publisher-specific licenses (i.e. ACS-specific) as well as “implied OA” when there is an evidence that an OA license of some kind was used, but it is not reported directly on the webpage at this location.

### Scope of the analysis and limitations

Articles from dates other than the ones specified were not considered (even if PubMed includes some out-of-date articles in its results). Only articles with a DOI were taken into account, and among them, there was a proportion not recognized by Unpaywall and thus, also not considered. Hence, the exclusion criteria after Unpaywall analysis includes out-of-date and those not scanned by Unpaywall (including papers without DOI).

Also, the Unpaywall system indexes thousands of institutional and subject repositories, but there are some still missing, and the database updates periodically, so some data might have changed.

Finally, the comparison with SARS CoV-1 and MERS CoV includes certain limitations such as differences in infection or death rates (especially with MERS). Likewise, the compared period times can also be a limitation, although this comparison is useful to demonstrate the huge current production.

## Results

### COVID-19 and SARS CoV-2 pandemic publications

**Figure 1.**
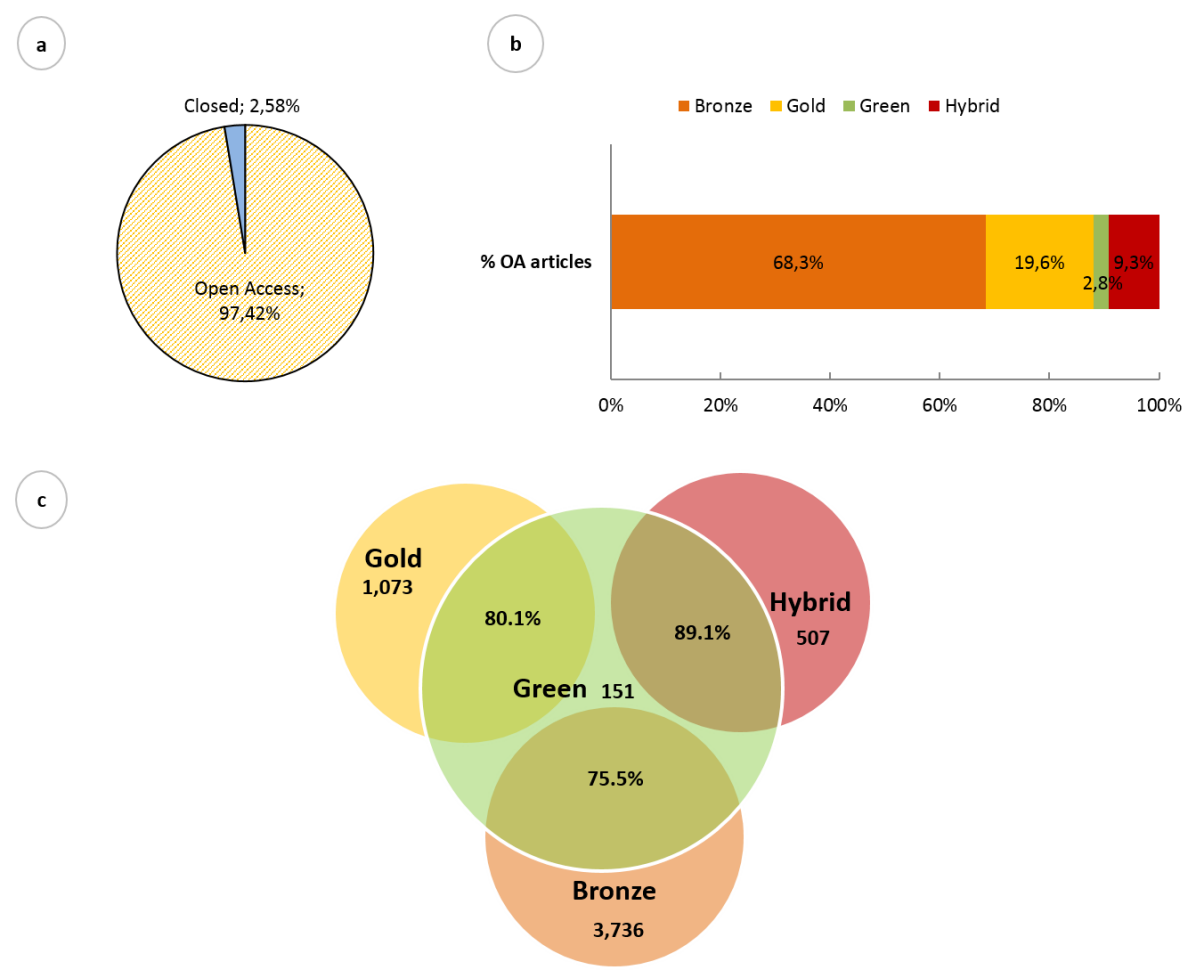
PubMed-hosted SARS CoV-2 related papers published in the first quarter (Q1) of 2020 and their Open Access (OA) information. ( **a**) Proportion of OA papers published during SARS CoV-2 pandemic. ( **b**) Percentage of publications divided by their OA publishing mode. ( **c**) Number of copies present in different repositories of each OA typology. (Data extracted from PubMed: 23 ^rd^ April 2020).

The data obtained about SARS CoV-2 from January 1 ^st^ to April 23 ^rd^ 2020 are shown in Figure 1. In total, 6,223 articles were retrieved from PubMed. Of these 9 were from 2019, 182 did not have a DOI assigned and 420 were not recognized by Unpaywall, and so were excluded from analysis; therefore, analysis was performed on a total of 5,612 articles.

When analysed, we observed that the number of articles published during the selected period increases daily. Figure 1a shows that 97.4% (n=5,467) of articles were published as OA. Regarding the type of OA, 68,3% (n=3,736) are classified as Bronze OA, followed by Gold OA (19.6%), Hybrid (9.3%), and Green OA (2.8%) ( Figure 1b). It is important to mention that 78.5% of the OA papers (n=4,294) have a copy in a repository, even if they are Gold (80.1%), Hybrid (89.1%) or Bronze (75.5%), which are known as *shadowed Green documents* ^12^ ( Figure 1c).

**Figure 2.**
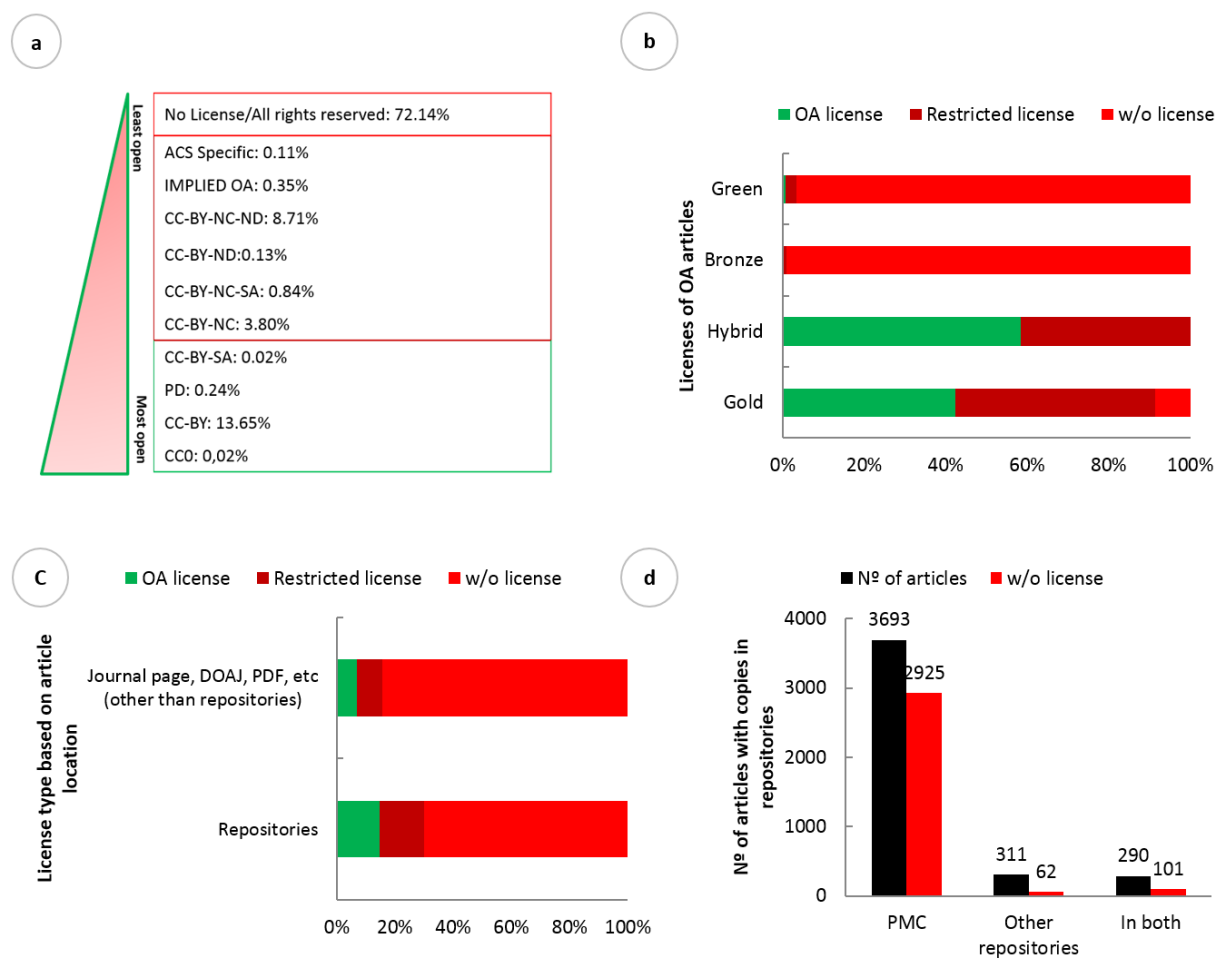
Licensing of Open Access (OA) SARS CoV-2 related papers hosted in PubMed first quarter (Q1) of 2020. ( **a**) Distribution of papers based on license category. Licenses were divided as: CC0, CC-BY, PD, CC-BY-SA, Implied OA, CC-BY-NC, CC-BY-NC-SA, CC-BY-ND, CC-BY-NC-ND, and those without any particular license. ( **b**) Distribution of papers with OA license (CC0, CC-BY, PD and CC-BY-SA), restricted license (Implied OA, CC-BY-NC, CC-BY-NC-ND, CC-BY-NC-SA, CC-BY-ND and ACS Specific) or without a license of each OA typology. ( **c**) License type (open, restricted or absent) attending each manuscript location: repository or non-repository (journal page, DOAJ or PDF). ( **d**) Number of papers present in PubMed Central (PMC), other repositories or at both locations, together with the number of these lacking any license. (Data extracted from PubMed: 23 ^rd^ April 2020).

In order to deeply analyse the OA situation, we also reviewed license information of all the OA papers. Figure 2 shows that most of these articles lack a license (72.1%). Most open licenses (CC0, PD, CC-BY and CC-BY-SA) are present in 13.9% of the papers, while the most restrictive ones (CC-BY-NC, CC-BY-ND, CC-BY-NC-SA and CC-BY-NC-ND), are represented in the same proportion (13.9%) of all the considered OA papers ( Figure 2b). Publisher implied licenses (named as “implied OA”) are included as the most restrictive ones as the majority of these are tied to the CC-BY-NC-ND one. Attending to each OA category, as expected, 99% of all Bronze papers don’t carry any license, with exception of 23 articles present at different repositories ( Figure 2b). It is remarkable that 93 of the articles classified as Gold OA (8.7%) don’t bear any license, even if they are published at an OA journal.

Related to this licensing section, when the repository copies were analysed, we observed that these copies carry a greater number of licenses (n=1,285, the 30% of all repository copies) compared to the ones located at any other source (i.e. journal page or free PDF; n=185, 15.7% of papers at these locations) ( Figure 2c). More precisely, 86% of these copies are located at PubMed Central (PMC) (n=3,693) with a 79.2% of them lacking a specific license ( Figure 2d). A total of 311 papers are located in other repositories different to PMC but in this case, more than 80% do have a specific licence. At this point is important to mention that after a deeper research of articles located at PMC we have observed the presence of articles that come from journals with an explicit license on its page that do not maintain it in PMC, where there is no reflected license other than a notice from each publisher stating that “access to these papers is temporary” (see Box1).

**Box 1.**

**Springer:** *“This article is made available via the PMC Open Access Subset for unrestricted research re-use and secondary analysis in any form or by any means with acknowledgement of the original source. These permissions are granted for the duration of the World Health Organization (WHO) declaration of COVID-19 as a global pandemic.”*

**Wiley:** *“This article is being made freely available through PubMed Central as part of the COVID-19 public health emergency response. It can be used for unrestricted research re-use and analysis in any form or by any means with acknowledgement of the original source, for the duration of the public health emergency.”*

**Elsevier:** *“Since January 2020 Elsevier has created a COVID-19 resource centre with free information in English and mandarin on the novel coronavirus COVID-19. The COVID-19 resource centre is hosted on Elsevier Connect, the company’s public news and information website. Elsevier hereby grants permission to make all its COVID-19-related research that is available on the COVID-19 resource centre – including this research content – immediately available in PubMed Central and other publicly funded repositories, such as the WHO COVID database with rights for unrestricted research re-use and analysed in any form or by any means with acknowledgement of the original source. These permissions are granted for free by Elsevier for as long as the COVID-19 resource centre remains active.”*

**Figure 3.**
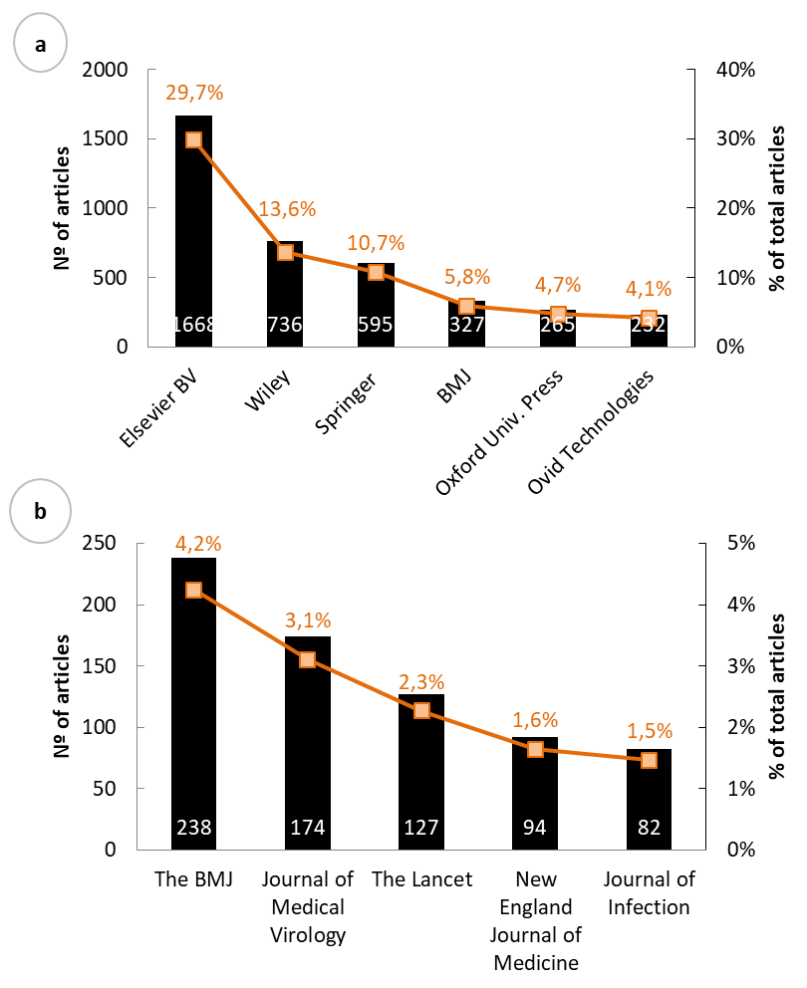
Publishers and journals that published the highest number of COVID-19-related papers hosted in PubMed in Q1 of 2020. Number and percentage of total publications distributed by most frequent publishers ( **a**) and journals ( **b**). (Data extracted from PubMed: 23 ^rd^ April 2020).

Furthermore, the most frequent publishers and journals during this period in relation to SARS CoV-2 were studied. The most frequent publisher is Elsevier, who published ~30% of papers, followed by Wiley (13.1%) and Springer (10.6%) ( Figure 3a, note that the remaining 31.9% not showed includes publishers with lower proportion than the ones shown). In terms of journals, *The British Medical Journal* (The BMJ), *Journal of Medical Virology* and *The Lancet* are those with the largest number of papers: 4.2, 3.1 and 2.3% of all analysed papers, respectively ( Figure 3b, representing the 6 most frequent journals).

**Figure 4.**
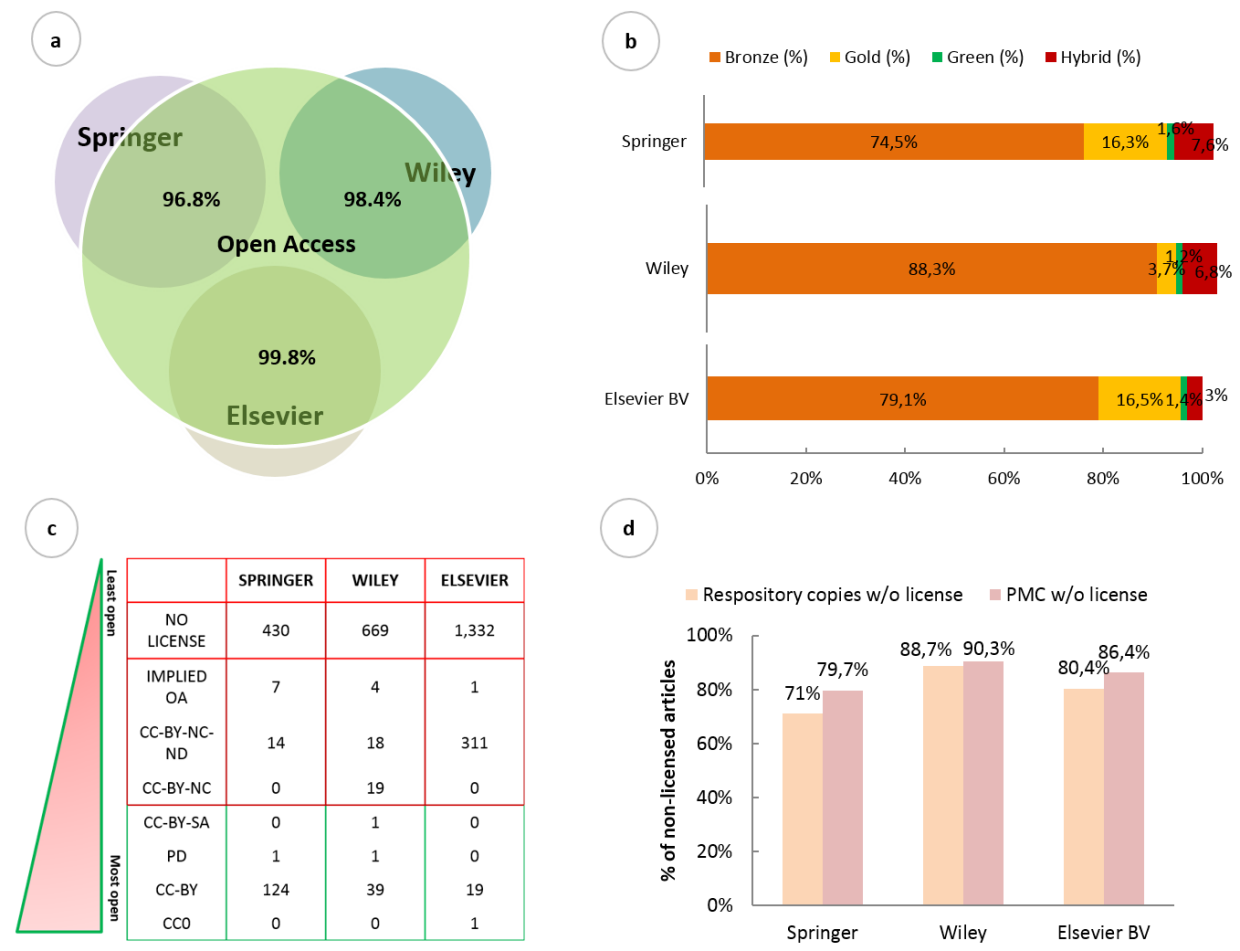
Analysis of the three most frequent publishers with more Open Access (OA) COVID-19 papers hosted in PubMed in Q1 of 2020: Elsevier, Wiley and Springer. ( **a**) Percentage of OA publications of the most relevant publishers: Elsevier, Wiley and Springer. ( **b**) Distribution of their open content by Gold, Hybrid, Green or Bronze status. ( **c**) Distribution of the licensed and non-licensed articles of the three publishers. ( **d**) Distribution of the non-licensed copies hosted in all repositories found (in salmon) versus the ones found only in PMC (in pink). (Data extracted from PubMed: 23 ^rd^ April 2020).

Based on these results, we specifically studied the COVID-19-related articles published by Elsevier, Wiley and Springer ( Figure 4). All three publishers release almost all SARS CoV-based articles as OA: Elsevier: 99.8%, Wiley: 98.4% and Springer: 96.8% of their published papers about the topic in the studied period ( Figure 4a). All three publishers publish the majority of their papers as Bronze OA ( Figure 4b), being Wiley the one with the highest proportion, with 88.3% of its OA manuscripts (n=663). Regarding Gold OA, Elsevier and Springer have published 16.5 and 16.3%, respectively, of their COVID-19 related articles under this category, a higher proportion compared to Wiley with only 3.7% (n=28). Looking at licensing, most of the OA publications from these publishers lack a license ( Figure 4c). At this point Wiley is the one with the highest number of papers without any license (89.1%, n=669) compared to Elsevier (80%, n=1,332) and Springer (74.7%, n=430) matching in each case with the corresponding number of Bronze articles. On the other hand, Springer is the one with more licensed papers (25.3%, n=146 licenses) and moreover, most of them are under CC-BY (n=124) ( Figure 4c)

Attending the copies located in different repositories, Elsevier publishes a copy of 99.3% of its manuscripts in different repositories, compared to Wiley (79.9%) and Springer (83.5%), and being in all cases PMC the most used one: more than 87% of these copies are in PMC. At the same time, when looked at the licensing, it is notable that there is a big proportion of copies in the three publishers lacking licenses, being again the PMC-hosted ones those with the fewest number: 79.7% for Springer, 90.3% for Wiley and 86.4% for Elsevier ( Figure 4d). As mentioned, for papers that are not shared with a specific license some publishers have decided instead, to show a specific note specifying their temporary access to their papers (see Box1).

### Publications about other coronaviruses and epidemics: SARS CoV-1 and MERS CoV

In order to compare the scientific production and OA publication during global health emergencies, both SARS CoV-1 and MERS CoV-related articles were studied using the PubMed database.

**Figure 5.**
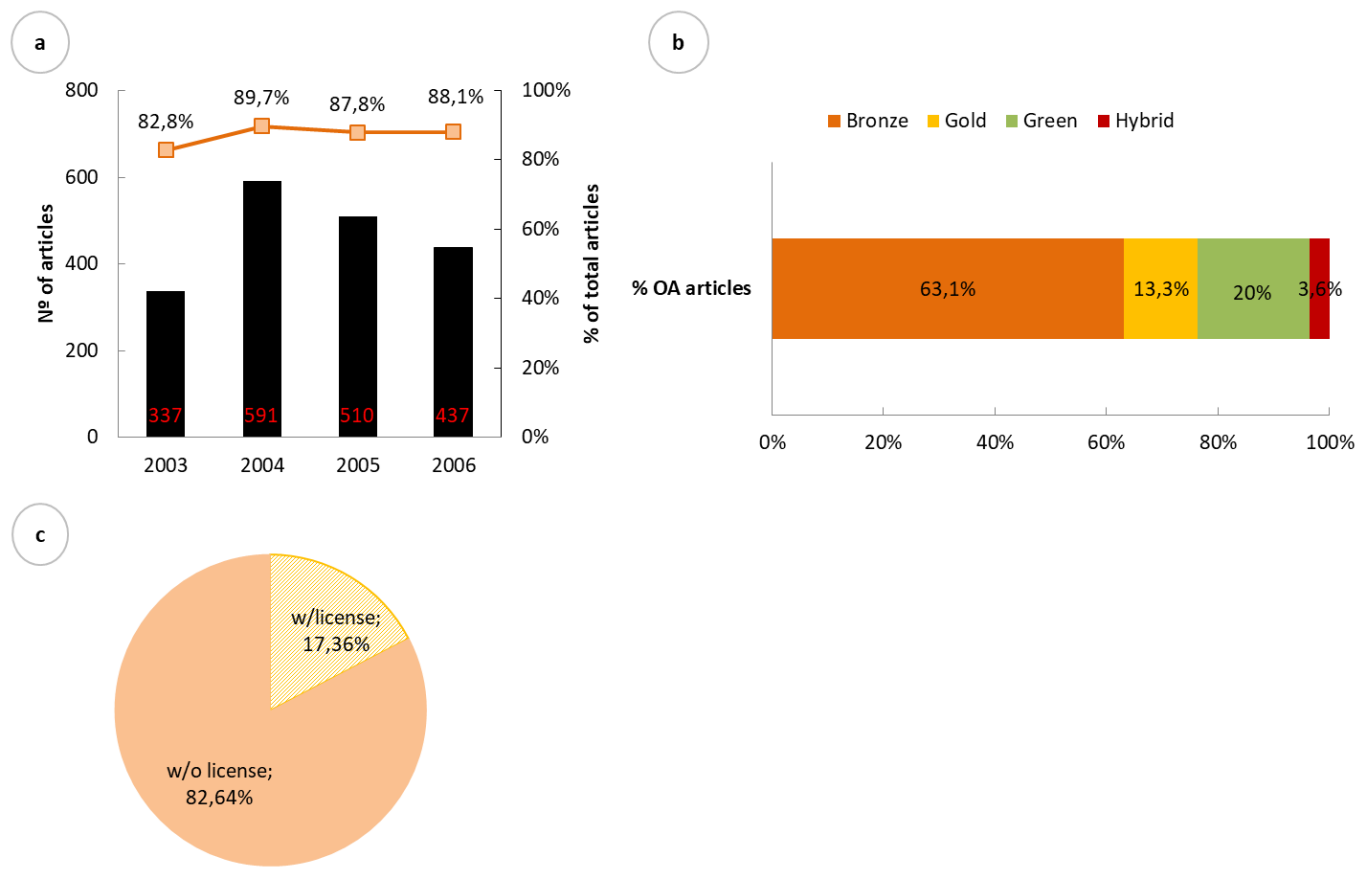
Publications related to SARS CoV-1 epidemic hosted in PubMed from 2003 to 2006 and their Open Access (OA) indicators. ( **a**) Number of total and OA publications about SARS CoV-1 epidemic during the first 4 years from the start of the epidemic. ( **b**) OA category of the OA published articles. ( **c**) Proportion of licensed and unlicensed OA articles. (Data extracted from PubMed: 19 ^th^ April 2020).

In the case of the SARS CoV-1 (Severe Acute Respiratory Syndrome CoronaVirus-1) epidemic, the first case was discovered in China during November 2002 ^15^. We therefore analysed publications published in 2003, 2004, 2005 and 2006 ( Figure 5). For the period from 2003 to 2006, PubMed returned a total of 2,396 articles, of which, after exclusion criteria, 1,875 were considered (476 lacked DOI and 45 were out-of-date). There was an increase in the number of publications from 2003 to 2004, with a decline onwards. The percentage of OA publications increased from 82 to 89% in the first year, maintaining a stable average of 87.6% throughout the analysed period ( Figure 5a). Among these open articles, 63.1% were published as Bronze OA, 20% as Green OA, 13.3% as Gold OA, and 3.6% in hybrid journals ( Figure 5b). About licensing, 82.6% (n=1,357) of the OA articles don’t carry any license (from which 24 are Gold OA), and from the licensed 17.3% (n=285), 10.7% (n=176) bear a CC-BY one ( Figure 5c).

**Figure 6.**
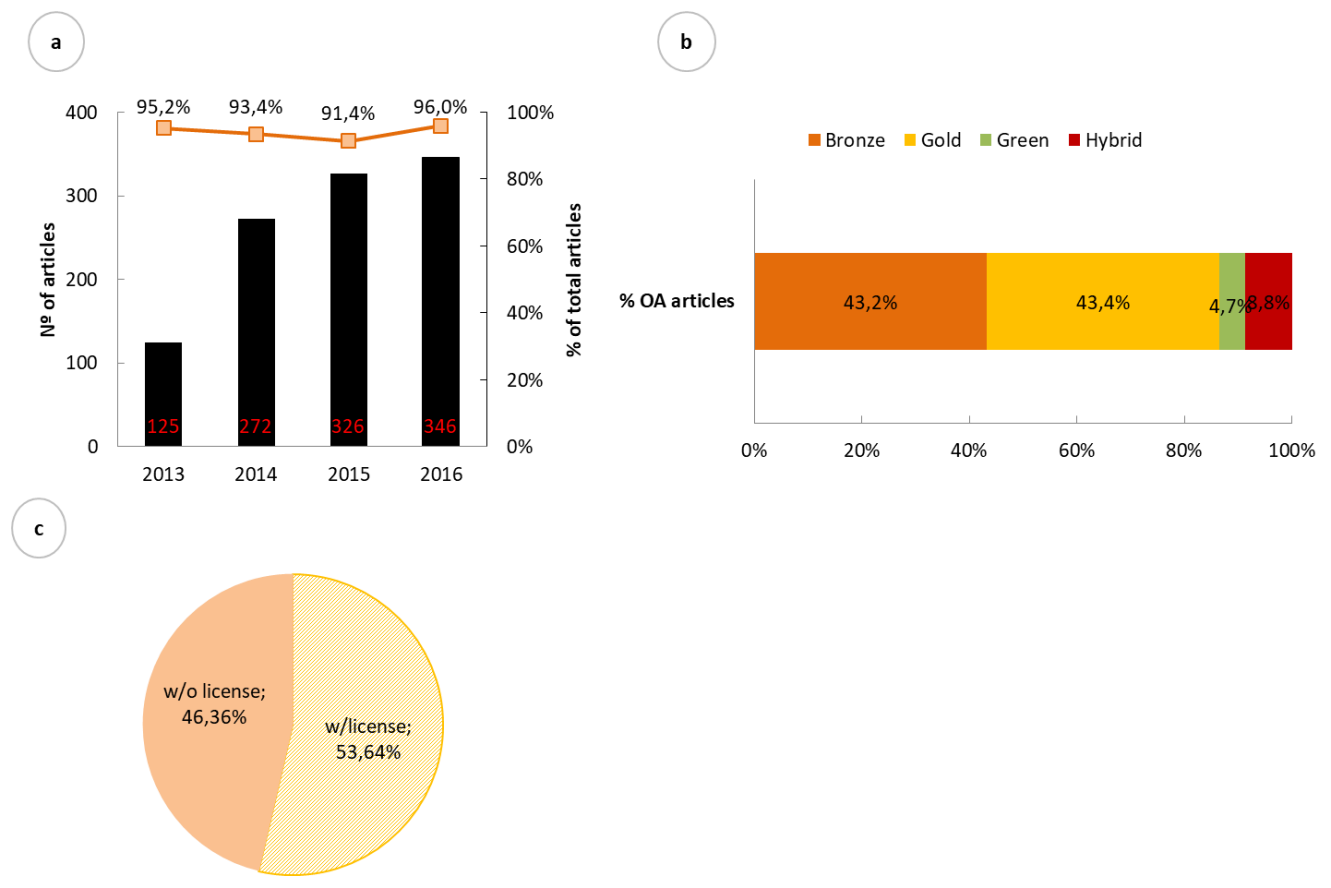
Publications related to MERS CoV epidemic and hosted in PubMed from 2013 to 2016 and their Open Access (OA) indicators. ( **a**) Number of total and OA publications based on the MERS CoV epidemic during the first 4 years from the epidemic outbreak. ( **b**) OA category of the OA published articles. ( **c**) Proportion of licensed and unlicensed OA articles. (Data extracted from PubMed: 19 ^th^ April 2020).

Next we performed the searches for the MERS CoV (Middle East Respiratory Syndrome Coronavirus) epidemic, whose outbreak began in September 2012 in Saudi Arabia ^16^. A total of 1,069 papers were obtained for the specified period (2013 to 2016). In this case, this number is significantly lower than the one found for SARS CoV-1 over time. In 2016, the year in which most papers are registered (n=346), the percentage of these published as OA remains constant and is very high, with an average of 93.8% ( Figure 6a). Unlike SARS CoV-2 and SARS CoV-1, 43.4% of MERS-related OA publications were published as Gold ( Figure 6b). Almost half of the OA articles have a proper license, and among them 31% carry a CC-BY one (n=307) ( Figure 6c). From the 46.4% of non-licensed articles, 23 are Gold, corresponding to the 5.3% of Gold OA articles.

As the goal is to focus on COVID-19, publishers’ and journals’ analysis has not been included for SARS CoV-1 nor MERS CoV searches as we do not consider relevant for our conclusions.

**Figure 7.**
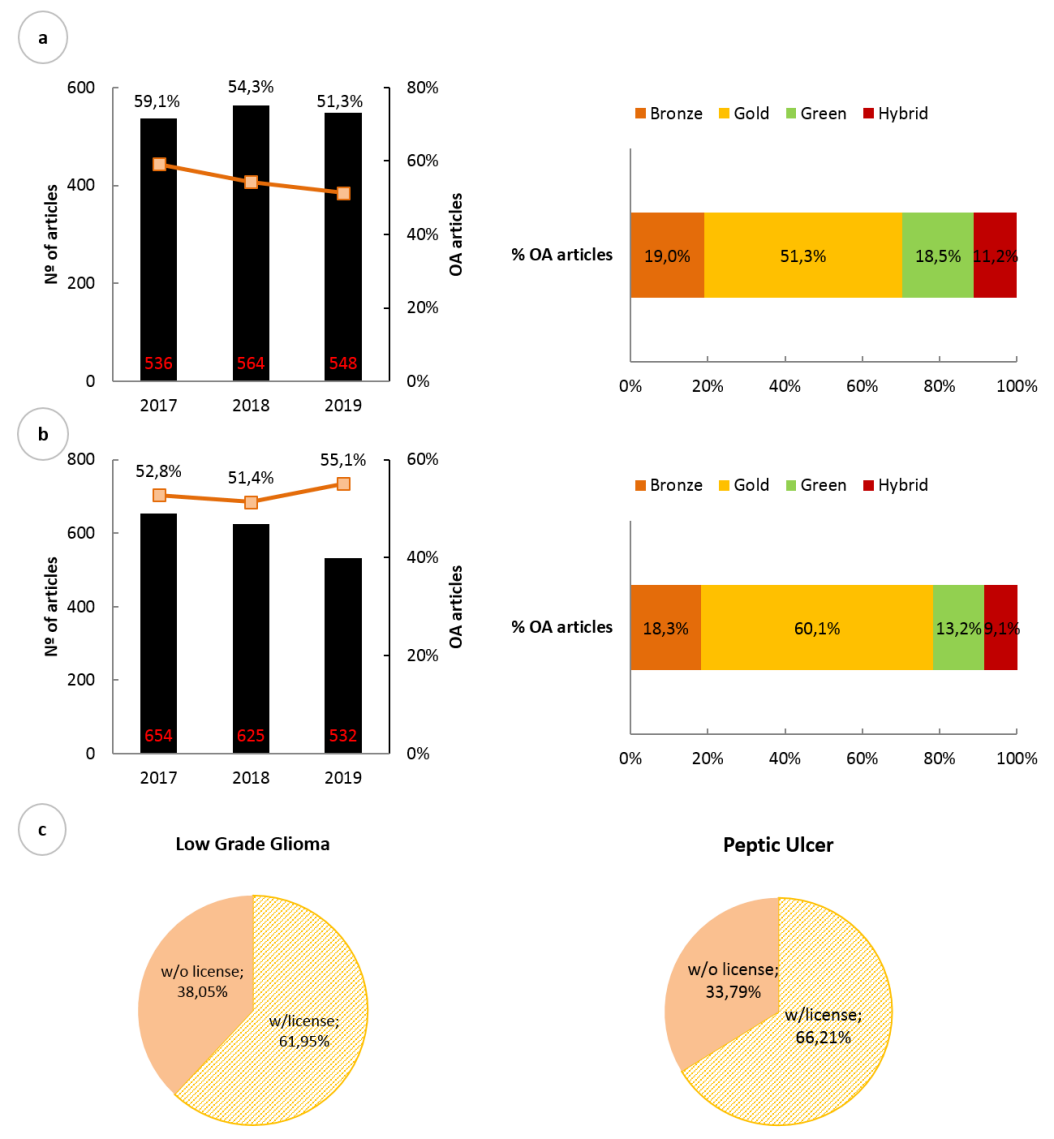
Analysis of the number and OA properties of papers about two chronic diseases: low grade glioma and peptic ulcer. Number of publications, OA proportion and category of articles related to low grade glioma ( **a**) and peptic ulcer ( **b**) during 2017, 2018 and 2019. ( **c**) Proportion of licensed and unlicensed OA articles of both diseases. (Data extracted from PubMed: 20 ^th^ April 2020).

In order to determine if these results are a consequence of the current extraordinary circumstances, a control of the research was established through the analysis of open content of chronic diseases considered constant over time. We performed searches for “low grade glioma” and “peptic ulcer”, which harbour similar output levels compared to SARS CoV-1 and MERS, obtaining a constant OA proportion for each case over the last 3 years ( Figure 7). This rate is low for all cases, with an average of 54.9% and 53% for low grade glioma ( Figure 7a) and peptic ulcer ( Figure 7b), respectively. In addition, articles concerning both diseases were mostly published as Gold OA, with a 51.3% and 60.1% of the OA articles in each case ( Figure 7a and 7b). Attending to licensing, the proportion of licensed papers is 61.9% for low grade glioma and 66.21% for peptic ulcer ( Figure 7c). Moreover, as a result of the high number of Gold OA papers, the proportion of CC-BY licenses is high for both cases (36.7% and 29.4%). It is important to underline that the number of repository copies for both controls represents 87.5% (n=791) and 77.7% (n=745) of the OA papers for low grade glioma and peptic ulcer, respectively.

## Discussion and conclusion

Compared to other emergency crises such as SARS CoV-1 or MERS CoV epidemics, the number of published papers during the current COVID-19 pandemic is huge. Our study (based only on the PubMed database) reveals that in only four months, the number of these articles is 17-times more than the number of documents available in the first year in the case of SARS CoV-1, and 48-times in the case of MERS CoV. A likely shortening of acceptance rates by journals is giving rise to information overload both for the scientific community but also for society, making it difficult to ascertain what really has a significant scientific value and as a consequence may affect decision-making.

In addition to the massive scientific production, after the pandemic declaration, publishers have made, not only COVID-19 but also previous SARS CoV-1 and MERS CoV related papers, openly available. From our study, both SARS-like viruses share the same limited conditions, i.e. are Bronze OA articles. On the contrary, a large number of MERS CoV-related papers present as Gold OA, suggesting high public funding from funders with OA policies during this period. In this context, it is surprising that there is a considerable proportion of Gold OA articles without licenses for all three diseases, which raises some uncertainties about whether some journals should still be listed in the DOAJ.

One of the main conclusions is that while Gold OA makes papers available immediately by the publishing journal itself, the predominant Bronze OA category, found by the present study, means that papers are freely hosted on publisher websites, without a license at all. Little is discussed in the OA literature about this category, but what is clear is that articles under this group without a categorised license do not allow extended reuse rights beyond reading. Thus, this “open” label removes rights to share or redistribute and, moreover, the publisher can revoke this access at any time. For instance, publishers state in their newly created coronavirus information centers or alike, about their temporary fee drop on coronavirus-related research, limited only to the duration of the crisis ( Springer Nature or Elsevier).

Green OA levels are low in PubMed-hosted COVID-19 papers compared to past outbreaks – especially during MERS CoV. A further analysis comparing those repositories that are contributing to access would be appropriate to determine this increase but at this level it is not possible to directly compare the levels of Green Open Access across these outbreaks. In this context, the number of COVID-19 articles that have a copy included in a repository almost reach 80% of OA papers. Although this data are similar to the ones found for SARS CoV-1, MERS and established controls, there is a difference in the proportion of licensed papers, and more specifically CC-BY licenses, that make corona-related papers less re-usable. In this regard, is of relevance PMC's role as the main repository where the vast majority of publishers have deposited a copy of their articles. This centralized inclusion of COVID-19 related papers is a positive issue for the scientific community. However, the fact of the restricted licensing used by many of the publishers where sharing and reuse is limited, together with the time period limitation, points out the weaknesses and opportunism of this model. Another point to highlight, as defined by Piwowar *et al.* ^12^, is the role of the non-OA journals (hybrid). The fact that these journals transiently give access to the reading of their articles (without any other use), benefit them from greater citation. It is not surprising that during this emergency situation, they are attracting the attention and curiosity of the entire world, including not only the scientific community but also non-scientific, increasing the citations and so the journals’ reputation and impact factor. This, together with the agreement with PMC to use its platform as the main COVID-19-repository, makes all this great opportunity to promote their reputation.

What is most interesting about the effect of the COVID-19 emergency on scientific research disclosure is what it says about the current publication model: it fails when a critical need arises for fast data dissemination. Our analysis elucidates that the current practice that is in use falls short of expectations of being the best model. We use the license as an heuristic to tell that this fast “opening” lacks basic OA principles, which are required in order to be transparent and, reusable. This could also have an important impact on a possible scenario where new outbreaks occur in the coming months or years.

We finally reflect that it seems clear that all stakeholders agree that Science only works when knowledge is shared. This unique and exceptional pandemic situation gives the opportunity to analyse the current publishing system in order to start new ways of scholarly communication, in a way that benefits the whole community, both researchers and society at large. This study has presented a part of Open Science-related issues and hopefully stimulates further research from the OA community regarding the use of Bronze OA and hybrid journals.

## Data availability

### Underlying data

Zenodo: “Open Access of COVID-19 related publications in the first quarter of 2020: a preliminary study based in PubMed” ^17, 18^ The paper reports only the filtered data. The underlying raw data (Excel data file containing Unpaywall results of each research query) are available in Zenodo:

Version 1: http://doi.org/10.5281/zenodo.3826038 under the terms of the Creative Commons Attribution 4.0 International license (CC-BY 4.0) ^17^.

Version 2: http://doi.org/10.5281/zenodo.3959950 under the terms of the Creative Commons Attribution 4.0 International license (CC-BY 4.0) ^18^.

## References

[1] Coronavirus Open Acess Letter. Accessed May 5, 2020. Reference Source

[2] Blair C: Request for Information: Public Access to Peer-Reviewed Scholarly Publications, Data and Code Resulting From Federally Funded Research.2020 Reference Source

[3] UNESCO - United Nations Educational Scientific and Cultural Organization: Science for Society. Accessed May 24, 2020. Reference Source

[4] Shanmugaraj B Siriwattananon K Wangkanont K: Perspectives on monoclonal antibody therapy as potential therapeutic intervention for Coronavirus disease-19 (COVID-19). *Asian Pac J Allergy Immunol.* 2020; 38(1):10–18. 10.12932/AP-200220-0773 32134278

[5] Rothan HA Byrareddy SN: The epidemiology and pathogenesis of coronavirus disease (COVID-19) outbreak. *J Autoimmun.* 2020;109:102433. 10.1016/j.jaut.2020.102433 32113704 PMC7127067

[6] Ahn DG Shin HJ Kim MH: Current status of epidemiology, diagnosis, therapeutics, and vaccines for novel coronavirus disease 2019 (COVID-19). *J Microbiol Biotechnol.* 2020;30(3):313–324. 10.4014/jmb.2003.03011 32238757 PMC9728410

[7] Falagas ME Pitsouni EI Malietzis GA: Comparison of PubMed, Scopus, Web of Science, and Google Scholar: strengths and weaknesses. *FASEB J.* 2008;22(2):338–342. 10.1096/fj.07-9492lsf 17884971

[8] Torres-Salinas D: Ritmo de crecimiento diario de la producción científica sobre Covid-19. Análisis en bases de datos y repositorios en acceso abierto. *El Prof la Inf.* 2020;29(2). 10.3145/epi.2020.mar.15

[9] He J Li K: How comprehensive is the PubMed Central Open Access full-text database?In: *IConference 2019 Proceedings* iSchools;2019 10.21900/iconf.2019.103317

[10] Else H: How Unpaywall is transforming open science. *Nature.* 2018;560(7718):290–291. 10.1038/d41586-018-05968-3 30111793

[11] Singh Chawla D: Half of papers searched for online are free to read. *Nature.* 2017 10.1038/nature.2017.22418

[12] Piwowar H Priem J Larivière V: The state of OA: A large-scale analysis of the prevalence and impact of Open Access articles. *PeerJ.* 2018;6:e4375. 10.7717/peerj.4375 29456894 PMC5815332

[13] Robinson-Garcia N Costas R Van Leeuwen TN: Open Access Uptake by Universities Worldwide. Accessed July 17, 2020. 10.7717/peerj.9410 32714658 PMC7353915

[14] Creative Commons: Creative commons license spectrum.svg - Wikimedia Commons. 2016; Accessed May 5, 2020. Reference Source

[15] Cleri DJ Ricketti AJ Vernaleo JR: Severe Acute Respiratory Syndrome (SARS). *Infect Dis Clin North Am.* 2010;24(1):175–202. 10.1016/j.idc.2009.10.005 20171552 PMC7135483

[16] Zaki AM Van Boheemen S Bestebroer TM: Isolation of a novel coronavirus from a man with pneumonia in Saudi Arabia. *N Engl J Med.* 2012;367(19):1814–1820. 10.1056/NEJMoa1211721 23075143

[17] Arrizabalaga O Otaegui D Vergara I: Open Access of COVID-19 related publications in the first quarter of 2020: a preliminary study based in PubMed. Published online May 14, 2020. 10.5281/ZENODO.3826038 PMC7438966 32850121

[18] Arrizabalaga O Otaegui D Vergara I: Open Access of COVID-19 related publications in the first quarter of 2020: a preliminary study based in PubMed. Published online May 14, 2020. 10.5281/ZENODO.3959950 PMC7438966 32850121

