# Supplementary material for: Open Access of COVID-19-related publications in the first quarter of 2020: a preliminary study based in PubMed
Source: F1000Res. 2020 Aug 12;9:649. Originally published 2020 Jun 26. [Version 2] doi: 10.12688/f1000research.24136.2 (PMC7438966; doi:10.12688/f1000research.24136.2)
Supplement: Peer review history

# Peer review: Reviewer response for version 2

**DOI:** 10.5256/f1000research.28399.r69296

**Published:** 2020-08-19

**Authors:** Cameron Neylon

**Affiliations:** 1 Centre for Culture and Technology, Curtin University, Perth, WA, Australia

The authors have addressed my concerns, including those that involves slight discrepancies in my reanalysis of the data. I have no further concerns and am happy to recommend this be certified as fully peer reviewed.

Is the work clearly and accurately presented and does it cite the current literature?

Yes

If applicable, is the statistical analysis and its interpretation appropriate?

Partly

Are all the source data underlying the results available to ensure full reproducibility?

Partly

Is the study design appropriate and is the work technically sound?

Partly

Are the conclusions drawn adequately supported by the results?

Partly

Are sufficient details of methods and analysis provided to allow replication by others?

Partly

Reviewer Expertise:

research evaluation, open access analysis

I confirm that I have read this submission and believe that I have an appropriate level of expertise to confirm that it is of an acceptable scientific standard.

# Peer review: Reviewer response for version 2

**DOI:** 10.5256/f1000research.28399.r69295

**Published:** 2020-08-17

**Authors:** Jonathon Alexis Coates

**Affiliations:** 1 University of Cambridge, Cambridge, UK

In version 2, Arrizabalaga *et al.* have appropriately addressed all of my concerns from the original version of the manuscript. I have no further concerns or comments and would approve for publication.

Is the work clearly and accurately presented and does it cite the current literature?

Partly

If applicable, is the statistical analysis and its interpretation appropriate?

Partly

Are all the source data underlying the results available to ensure full reproducibility?

Yes

Is the study design appropriate and is the work technically sound?

Yes

Are the conclusions drawn adequately supported by the results?

Yes

Are sufficient details of methods and analysis provided to allow replication by others?

Partly

Reviewer Expertise:

Metaresearch, preprints

I confirm that I have read this submission and believe that I have an appropriate level of expertise to confirm that it is of an acceptable scientific standard.

# Peer review: Reviewer response for version 1

**DOI:** 10.5256/f1000research.26624.r65638

**Published:** 2020-08-04

**Authors:** Pilar Rico-Castro

**Affiliations:** 1 Fundación Española para la Ciencia y la Tecnología (FECYT), Madrid, Spain

This work addresses how the recent COVID-19 pandemic has boosted open access practices among publishers and researchers, and it concludes that current practices include neither proper nor adequate licensing of research articles by publishers. Despite the fact that publishers comply with OA compromises with authors, they do not meet with larger open science requirements – especially those regarding scientific contents’ reusability. This paper opens up a very necessary discussion about the role that publishers can play as enablers or avoiders for making scientific knowledge findable, accessible, reusable, and interoperable, even when they fulfill formal open access requirements. The analysis is made for the early months of the COVID-19 pandemic, a time in history in which humans have been confronted with a vital need of scientific responses for their simplest every day routines. This extreme circumstance is used to light the real dimension of our dependence from open science, not only as researchers but as human beings, and to point each actor’s responsibility in providing the conditions for scientific advances to meet with the FAIR and the OS requirements.

 However, the work has a few weaknesses that need to be properly addressed.

**Major concerns:**

 1.- Need for clarification of the article’s objective and the research question. Under the “Introduction” section a variety of ideas are mixed and the research objective is not clearly stated. Mentions to the “scientific collaboration” with no previous context nor ulterior analysis, and sentences like “we wonder if the scientific community is ready to share and consume openly such information” contribute to blur the research objective. A clear statement on the research objective is needed.

 2.- Need for clarification of the research object. It is not clear whether or not pre-prints are included within the scope of the analysis. The paper needs an explicit declaration on that.

 3.- The comparative method has not been adopted correctly for the following reasons:

 - The comparability of the search “2019-nCoV OR 2019nCoV OR COVID-19 OR SARS-CoV-2 OR (Wuhan AND corona-virus)”, for which only articles published in a four months period has been considered, with the search “SARS CoV” OR “Severe Acute Respiratory Syndrome Coronavirus”, for which a three years period has been considered (2003 to 2006) and with the search “MERS CoV” OR “Middle East Respiratory Syndrome Coronavirus” for which a different three years period has been considered (2013 to 2016) needs a previous normalization. The time periods are very different (4 months vs. 3 years), and that invalidates the comparison. For sorting this out, the author could either normalize the data for all the periods analyzed to a "month unit", or use in their analysis only the first 4 months of all the health crises in comparison.

 - The authors are comparing an open period (this crisis is not yet over and we do not know how long it would last) with two closed crises. This should be acknowledged in the text as a methodological limitation.

 - The health crises under comparison contain large differences amongst them that require to be taken into consideration and to be acknowledged in the text as a methodological limitation. That the three situations have been classified as health emergencies is not enough for them to be comparable. They hold important differences regarding infection rates and death rates. The rapid spread of the recent pandemic has led governments all around the world to adopt never seen before very drastic measures (like lockdown) with a formidable impact on our economic system. Under this circumstance, a huge pressure has been put on the scientific community; therefore it has affected publication rates. In addition, the recent pandemic is taking place where the public debate about open access to scientific research is at its peak time. Many governments and funding agencies all around the world are launching OA policies (PlanS, as an example) and negotiating transformative agreements with large commercial publishers. All these conditions have a strong potential to affect OA availability of publications, both regarding publishers’ editorial practices and researchers’ publication patterns, thus affecting the comparison levels of the different periods considered in the analysis. All these elements should be acknowledged as difficulties for the comparison in the paper.

 4.- Unpaywall categories are not mutually-exclusive. This should be properly addressed and explained in the analysis. A publication can be Gold and Green OA simultaneously, and it can also be Hybrid and Green OA simultaneously. Moreover, Bronze category can be combined with each of the remaining three categories (Green, Gold, and Hybrid) as well as with the Gold-Green and Hybrid-Green combinations. The only ones that are mutually-exclusive are Gold and Hybrid categories. This opens a major methodological concern: whether data have been double counted or not. Therefore:

 - What compatibilities exist between the different categories should be properly explained.

 - A clarification about whether or not there is double counting needs to be made.

 - In the case that double counting has been avoided, authors must explain from which category the items have been removed from, and under which criterion.

 - Authors do not explain how they found out that Green and Hybrid papers are classified under Bronze category. This explanation should be included under the results section.

 5.- Clarify the role of CC licenses within the OS requirements.

 The relationship between CC licenses and OS reuse requirements is not properly mentioned in the text. Brief explanations to clarify what CC licenses are and what role they play is needed.

 6.- Delete non-evidence based conclusions. The following sentences are not based in any proven evidence or data:

 - “From the data, it can be seen that the number of articles published during the selected period increases daily”. There are no data referring to daily publications in the paper.

 - “Shortening of acceptance rates by journals is giving rise to information overload both for the scientific community but also for society, making it difficult to ascertain what really has a significant scientific value and as a consequence may affect decision-making”. This cannot be inferred from the analyzed data. Nothing has been proven about the shortening of acceptance rates by journals or about the scientific value of the publications. None of these issues have been addressed in the paper.

 - “In addition to the massive scientific production, after the pandemic declaration, publishers have made, not only COVID-19 but also previous SARS CoV-1 and MERS CoV related papers, openly available”. This cannot be inferred from the analyzed data and it has not been proven. (Actually, in my opinion, the most likely explanation for finding SARS CoV-1 and MERS CoV related papers in OA is that the embargo period has already expired.)

 - “… as the majority of the documentation is not free all the time, the number of subscriptions might be affected since it is possible that new non-subscribed readers obtained during this pandemic period have read articles from these journals and want to continue doing it.” This cannot be inferred from the analyzed data and it has not been proven. Actually, it is quite unlikely since scientific journals’ subscriptions are not decided nor negotiated by researchers, but by academic libraries.

 - “What is most interesting about the effect of the COVID-19 emergency on scientific research disclosure is what it says about the current publication model: it fails when a critical need arises for fast data dissemination”. This sentence from the conclusion section goes against the evidence presented in the analysis since authors have shown that of a total of 5,611 published articles related to COVID-19 pandemic, 4,986 were in OA in some way or another. Also, nothing has been proven about the speed of dissemination; therefore no conclusions can be drawn about this issue.

 - “We finally conclude that it seems clear that all stakeholders agree that Science only works when knowledge is shared.” There is no evidence to sustain this sentence. It should be either proven or deleted.

 7.- Strength evidence-based conclusions:

 - “While Gold OA makes papers available immediately by the publishing journal itself, the predominant Bronze OA category, found by the present study, means that papers are freely hosted…” This whole paragraph contains the main evidence-based conclusion of the work. The idea that OA is not enough, and that despite the fact that publishers put a multitude of works in open access in response to a certain situation (in this case pandemic) it that does not guarantee an open, findable, accessible, interoperable and reusable science, should be a strength in the paper.

 - “Our analysis demonstrates that the current alternative that is in use falls short of expectations of being the best model, since this fast opening lacks basic OA principles, which are required in order to be transparent, reusable and…” This sentence contains the second main evidence-based conclusion of the work. It should be a strength in the conclusions section of the paper.

**Minor concerns:**

 1.- Need for brief definitions:

 - Definitions of Open Access and Open Science concepts as well as proper citations about both concepts are missing. Open Science means much more than Open Access. A proper brief definition of both concepts is needed for the reader not to mix them up.

 - “In order to analyse publications concerning COVID-19 and their level of openness, we have chosen PubMed instead of other multidisciplinary databases, like Web of Science (WoS) or Scopus”. Clarify in this sentence that PubMed, WoS, and Scopus are databases for bibliographic references.

 2.- Need for cites.

 - “In this unique context of the pandemic, publishers are announcing massive OA changes, primarily by making their corona-virus-related articles freely available through databases, such as PubMed Central, together with other public repositories”. This paragraph lacks proper citations and a more detailed explanation on the cited new practices launched by publishers that differentiates pre-print repositories from opening peer reviewed published articles.

 3 .- Need for web references of Scopus, PubMed, MEDLINE, and PubMed Central (PMC), as it has been done for WoS.

 4.- Correct the expression “five categories” because there are only four (Gold, Hybrid, Green, and Bronze).

 5.- Clarification of the meaning of “Q1” in Figures 1, 2, 3, and 4. It is confusing since the reader tends to think of the 1 ^st^ quartile of the JIF.  

 6.- Change Figure 1a since it is confusing. It is not straightforward to see that the top portion is a part of the bottom portion. It looks like the addition of both is the total. There are more appropriate figures to show both the total and its proportion in a more intuitive manner.

 7.- Clarify Figure 1c. Figure 1c needs further clarification in the text about the meaning of "Via page says license", and "Via free article" categories.

 8.- Mention why the publishers’ and journals’ analysis has not been made for SARS CoV-1 nor MERS CoV searches. The analysis conducted for the three periods is different. No description regarding neither publishers nor journals has been made for publications about SARS CoV-1 nor MERS CoV.

 9.- Completing data in Figure 3a. The percentages in graph 3a add up no more than 68.6%. This means that there are 31.4% of the publications that are not included in the graph. This is important to be noticed since the remaining 31.4% is a higher figure than the largest category represented (29.7%). It is recommended to include a category "others" with 31.4% of the publishers. The dispersion of the data is very large. Focusing the analysis only on Elsevier, Wiley and Springer is reducing it to 54% of the data. This should be mentioned it in the text.

 10.- Figure 3b refers exclusively to 12.7% of the data. This should be mentioned it in the text.

 11.- Explain graph 4b in the text.

 12.- Change graph 4d. This graph is not very accurate. I suggest using a similar graph than the previous one (4c).

 Finally, this paper opens the door for further research to be done in the future, like the analysis of the relationship between the four categories of OA (Gold, Hybrid, Green, and Bronze), the CC licenses that they use, and the publishing practices of the different large publishing companies. It would be fantastic if the authors continue their work in this way.

Is the work clearly and accurately presented and does it cite the current literature?

Partly

If applicable, is the statistical analysis and its interpretation appropriate?

Partly

Are all the source data underlying the results available to ensure full reproducibility?

Yes

Is the study design appropriate and is the work technically sound?

Partly

Are the conclusions drawn adequately supported by the results?

Partly

Are sufficient details of methods and analysis provided to allow replication by others?

Yes

Reviewer Expertise:

R&D policy making; Open Access; Open Science; research infrastructures; open repositories; peer reviewed journals; public policies

I confirm that I have read this submission and believe that I have an appropriate level of expertise to confirm that it is of an acceptable scientific standard, however I have significant reservations, as outlined above.

# Peer review: Reviewer response for version 1

**DOI:** 10.5256/f1000research.26624.r65637

**Published:** 2020-07-08

**Authors:** Jonathon Alexis Coates

**Affiliations:** 1 University of Cambridge, Cambridge, UK

**Summary:**

 Arrizabalaga *et al.* address the important issue of accessibility in biomedical publishing. Utilising data from Unpaywall the authors investigate the open access status of COVID-19 articles published within the first four months of 2020. The majority of the COVID-19 literature investigated in this study are classified as bronze open-access, potentially subject to removal behind a paywall at any time. There is also a comparison to other epidemics (SARS-Cov-1 and MERS) and more recent literature that enables some comparisons between the literature.

 There are inherent weaknesses to such a study due to the time period chosen which, due to the nature of the temporary open-access of many articles, is subject to change in the future. However, this is acknowledged by the authors in the text and can be further addressed through additional discussion. Overall, this is an important topic assessing the early phase of the pandemic with this study requiring some relatively minor changes.

**Major concerns:**

1. Clearer definitions for the different levels of open-access and licences, perhaps as a table. For those not familiar with the open-access terminology this would make the manuscript much clearer and easier to follow.
2. Better distinguish between open-access articles and those that are temporarily open-access through further discussion and analysis. The publisher motivations are highly important, particularly if a large proportion of the current bronze open-access subset is likely to be placed behind a paywall in the future.
3. Clear details on how data were collected, for example, was the data collected via the Unpaywall API or by a list of DOI's? This is particularly relevant for the date of collection, which will impact the results should others attempt to replicate as the authors themselves state in the limitations.
4. Fig. 1A is misleading, presenting all articles and the open access articles summed together. Data should be presented as a stacked bar not summing the articles with the open-access subset or as a Venn diagram. Moreover, Fig. 1C is confusing as currently displayed and may be better removed, with the information communicated in the text instead.
5. It would be nice to see the data for licences used for SARS-CoV-1, MERS, low grade glioma and peptic ulcers in the relevant figures. This is important information that helps to further understand the re-usability of open-access articles.

 

**Minor concerns:**

  

1. The number of preprints has increased dramatically as a means of sharing COVID-19 research. It may be useful for the authors to discuss this especially considering the limited nature of some of the open-access COVID-19 literature.
2. Licence “CC” should be “CC0” in text and figures throughout.
3. Clearer discussion over what the authors recommend as good open access principles (including the licence types).

Is the work clearly and accurately presented and does it cite the current literature?

Partly

If applicable, is the statistical analysis and its interpretation appropriate?

Partly

Are all the source data underlying the results available to ensure full reproducibility?

Yes

Is the study design appropriate and is the work technically sound?

Yes

Are the conclusions drawn adequately supported by the results?

Yes

Are sufficient details of methods and analysis provided to allow replication by others?

Partly

Reviewer Expertise:

Metaresearch, preprints

I confirm that I have read this submission and believe that I have an appropriate level of expertise to confirm that it is of an acceptable scientific standard, however I have significant reservations, as outlined above.

# Response

**Published:** 2020-07-27

**Authors:** Olatz Arrizabalaga

**Affiliations:** Instituto de Investigación Sanitaria Biodonostia, Spain

*Dear Jonathon,*

*Find here below, in red all the explanations to your helpful comments and insights.*

 Arrizabalaga et al. address the important issue of accessibility in biomedical publishing. Utilising data from Unpaywall the authors investigate the open access status of COVID-19 articles published within the first four months of 2020. The majority of the COVID-19 literature investigated in this study are classified as bronze open-access, potentially subject to removal behind a paywall at any time. There is also a comparison to other epidemics (SARS-Cov-1 and MERS) and more recent literature that enables some comparisons between the literature.

 There are inherent weaknesses to such a study due to the time period chosen which, due to the nature of the temporary open-access of many articles, is subject to change in the future. However, this is acknowledged by the authors in the text and can be further addressed through additional discussion. Overall, this is an important topic assessing the early phase of the pandemic with this study requiring some relatively minor changes.

*Thank you very much for your thoughtful overall evaluation. We do agree with all of your comments and we are addressing all them and recommendations in Version 2 of the paper*.

 Major concerns:

 Clearer definitions for the different levels of open-access and licences, perhaps as a table. For those not familiar with the open-access terminology this would make the manuscript much clearer and easier to follow.

*The authors agree completely with this perception. In the V2 of the paper we update these conclusions and added tables to some figures in order to follow easier the paper.*

 Better distinguish between open-access articles and those that are temporarily open-access through further discussion and analysis. The publisher motivations are highly important, particularly if a large proportion of the current bronze open-access subset is likely to be placed behind a paywall in the future.

*Totally agree, we have included more results and conclusions about this in the new version.*

 Clear details on how data were collected, for example, was the data collected via the Unpaywall API or by a list of DOI's? This is particularly relevant for the date of collection, which will impact the results should others attempt to replicate as the authors themselves state in the limitations.

*Yes you are right. Together with the new analysis we have updated the methodology section in order to clarify this issue.*

 Fig. 1A is misleading, presenting all articles and the open access articles summed together. Data should be presented as a stacked bar not summing the articles with the open-access subset or as a Venn diagram. Moreover, Fig. 1C is confusing as currently displayed and may be better removed, with the information communicated in the text instead.

*Changed in version 2.*

 It would be nice to see the data for licences used for SARS-CoV-1, MERS, low grade glioma and peptic ulcers in the relevant figures. This is important information that helps to further understand the re-usability of open-access articles.

*Yes, you are right. We have included this information in the version too.*

 Minor concerns:

 The number of preprints has increased dramatically as a means of sharing COVID-19 research. It may be useful for the authors to discuss this especially considering the limited nature of some of the open-access COVID-19 literature.

*Yes you are right. Although we mention it in some of the sections of the article, perhaps it would be a good idea to be able to make a deeper analysis just about it since it might be a topic that gives for a whole paper.*

 Licence “CC” should be “CC0” in text and figures throughout.

*Yes. It is CC0, It is update in the v2 or the paper.*

 Clearer discussion over what the authors recommend as good open access principles (including the licence types).

*Hope what is in the new version conforms this point.*

**

*Thank you so much for your great comments.*

# Peer review: Reviewer response for version 1

**DOI:** 10.5256/f1000research.26624.r65639

**Published:** 2020-07-06

**Authors:** Cameron Neylon

**Affiliations:** 1 Centre for Culture and Technology, Curtin University, Perth, WA, Australia

**Review and Replication Report for Arrizabalaga *et al. *(2020)**

 General Observations

 The paper addresses an important issue on a dynamic and moving subject, the availability of research on COVID-19 within the context of the pandemic. This is a useful and potentially important record of the state of the literature at a particular point in time. Its timeliness is also related to some of its weaknesses in terms of how the state of the relevant literature is changing. Nonetheless, it presents a useful record and, with some relatively minor alterations, will provide an important record of a moment in time.

 Recommendations for clarification

 There are a series of changes and clarifications I would recommend to the paper as the conclusions depend on the specificity of categories of open access referred to. It is important to be clear about the details of what is meant by categories such as 'hybrid' and 'bronze' and how closely these relate to the heuristics that are used to detect them, which are necessarily imperfect.

 Specifically, it is important to distinguish between the category of articles that are temporarily released by publishers from behind a paywall, and those articles that are detected by a process of identifying free copies on a publisher website without an explicit license ('bronze'). As the argument of the paper hinges on the identification and categorisation of these articles and implicitly on the motivations of publishers in releasing them it is critically important that the category of access models (promotional or emergency release) is distinguished from the categories that can be detected ('bronze').

 Specific suggested changes to address these and related issues:

1. Under 'Data analysis' it is not immediately clear to me why the Web of Science classification is referred to. I would argue that what should be presented is the detailed implementation of exactly how the categories are assigned in this article (see Replication report below for an example of this). If the categories provided by Unpaywall are used directly this should be explained.
2. More detail on the process of data preparation would be helpful. The provision of the finalised data is very useful but details of how the Unpaywall data was collected (via the API in OpenRefine or by upload of a set of DOIs?) and exactly when (because this makes a difference to analysis, see below).
3. Throughout the discussion, there is a potential for confusion with terms like 'non-licensed Bronze'. I would use 'Bronze' throughout, perhaps repeating the point that it is by definition non-licensed. Similarly the statement '...many of these Bronze OA publications have been published in Hybrid journals...' is confusing as by the definitions used here Bronze will always be in a hybrid journal.
4. A related issue is that I would prefer to explicitly use a term like 'DOAJ Gold' to refer to articles in purely open access journals as there is significant variation across the literature in the application of this term and being explicit throughout would help.

There is also some confusion in the description of Green OA. Specifically, the definition of Green adopted here is one that applies only to those articles that are not also Gold. This is standard practice, although I personally think it inadvisable, here it leads to significant confusion. In fact, the contribution of repository access to this corpus is nearly as great as that of publishers with 43% being described as "shadowed green". I would argue for a more detailed analysis of the repositories being used in the results section.

 In policy and analysis terms this is arguably as important a contribution to access as that of publishers. I would argue for a greater analysis of this part of the corpus (see replication report for further details). The choice of Pubmed Central to accept the deposit of articles with no guarantee of long-term access is a significant potential issue. This both raises questions about definitions of "green" open access and licensing that deserve a little more attention in the discussion in my view.

 The paragraph in the discussion that commences "In line with this..." is difficult to parse. It is not clear to me that the lack of a license on the publisher site (which results in categorisation as bronze) necessarily flows through to the licensing of the Pubmed Central version. This deserves further analysis (see below). The paragraph reads as though the assignment of only 3% to green implies that the repository copies are not guaranteed. My reading of the methodology does not agree with this. This strengthens the argument for an explicit description of the category assignment.

 Finally, I think the conclusion is probably too strong on what the analysis demonstrates vs what the concerns of the authors are. While I agree with their conclusion that it is unfortunate that the release of otherwise restricted content in the context of the pandemic has such limitations in terms of the time frame and re-use this analysis cannot show the downstream effects of those restrictions, which will need to await future analysis. I think a sharper distinction between the observations made and the concerns of the authors would benefit the article.

 Minor issues

 Figure 1 has a number of misleading characteristics. In Figure 1a a bar chart is presented that shows both *all* articles and the oa subset but adding the two together. Figure 1c is also confusing. As noted below I don't understand why the data has been divided up the way it has. Both the conflation of the two evidence types for which Unpaywall found free articles, combined with leaving out of DOAJ as evidence source for the second pie chart seems odd and these results are not used elsewhere in the paper. I would leave 1c out and use a Venn diagram for 1a and a bar chart for 1b Figure 2 and related text. The license category of 'cc' is presumably cc0.

 I find Figure 4d confusing. Would it not be better to show some quantitative parameter for each of the publishers rather than the -OPEN and +OPEN? Perhaps open licenses as a proportion of all articles or something similar?

 In analysing past outbreaks the issue of increases in repository-mediated (green) OA over time should be explicitly mentioned. This might particularly be included in a comparison of those repositories that are contributing to access. This does not directly affect the conclusions of these sections as the proportion of green is not otherwise interpreted but the potential for confusion means this should be at least mentioned with a statement saying that it is not therefore possible to directly compare the levels of green open access across these outbreaks.

**Replication Report**

 I report on a direct replication using the supplied data. Broadly speaking I confirm the overall results with some reservations and slight differences which are noted below. There seems little value in reproducing the Unpaywall data from the supplied DOIs. A manual search of PubMed could be used to confirm the numbers and identity of DOIs but I do not conduct that here at this point.

 The full code for the Replication report can be found at Github as a Jupyter Notebook at:

https://github.com/cameronneylon/replication_report_Arrizabalaga_2020

 And on Mybinder.org at:

https://mybinder.org/v2/gh/cameronneylon/replication_report_Arrizabalaga_2020/master

 Here I provide only the main points in summary. See Github for the fully worked analysis and code for comparison purposes.

 Minor issues

1. The dataset has 5621 rows of data, not 5611 as specified in the paper. Is this to do with blank entries or entries without DOIs?
2. There are 4989 oa articles by my analysis, not 4986 as specified in the paper. Comparison to the provided data provides 4991 oa articles, and the difference is explained by the two entries for which the JSON does not parse.
3. Not immediately clear why for Fig 1c the two categories of free articles have been combined?
4. Why in Figure 1c are the oa types reported only for those articles where the evidence type is either free article or free pdf? Why are the DOAJ evidence examples not included?
5. Figure 2. Slight variation in the percentages calculated from the dataset.
6. There are slight issues in 4b and 4c with the license assignment.
7. As noted in general comments I would drop Figure 4d as it is confusing and it is not clear to me that it is supported by the data where Wiley does not appear to have many more open licenses than Elsevier.

Major Issues

1. The numbers in the paper do not seem to correspond directly to those in the dataset provided
2. It appears the article does not use DOAJ as the criterion for gold but the is_oa_journal field from unpaywall. This explains the variance between my analysis and that in the article for "gold" as defined by my code (16% vs 19% using the data provided, vs 21.5% given in Figure 1).
3. In Figure 5-7 I think there may be an error in the counting of OA articles, counting all those articles for which there is an 'is oa' entry and not only those where it is set to True.

Is the work clearly and accurately presented and does it cite the current literature?

Yes

If applicable, is the statistical analysis and its interpretation appropriate?

Partly

Are all the source data underlying the results available to ensure full reproducibility?

Partly

Is the study design appropriate and is the work technically sound?

Partly

Are the conclusions drawn adequately supported by the results?

Partly

Are sufficient details of methods and analysis provided to allow replication by others?

Partly

Reviewer Expertise:

research evaluation, open access analysis

I confirm that I have read this submission and believe that I have an appropriate level of expertise to state that I do not consider it to be of an acceptable scientific standard, for reasons outlined above.

# Response

**Published:** 2020-07-27

**Authors:** Olatz Arrizabalaga

**Affiliations:** Instituto de Investigación Sanitaria Biodonostia, Spain

*Dear Cameron,*

*Find here below, in italics, all the explanations to your helpful comments and insights.*

**General Observations**

The paper addresses an important issue on a dynamic and moving subject, the availability of research on COVID-19 within the context of the pandemic. This is a useful and potentially important record of the state of the literature at a particular point in time. Its timeliness is also related to some of its weaknesses in terms of the how the state of the relevant literature is changing. Nonetheless it presents a useful record and, with some relatively minor alterations, will provide an important record of a moment in time.

*Thank you very much for your thoughtful overall evaluation. We do agree with this perception and we are addressing all your comments and recommendations in Version 2 of the paper, and we also underline the state of the changing status of the relevant literature about COVID-19 in order to contribute, with this piece, to the meta-research about so relevant topic in the current challenging context.*

**Recommendations for clarification,**

There are a series of changes and clarifications I would recommend to the paper as the conclusions depend on the specificity of categories of open access referred to. It is important to be clear about the details of what is meant by categories such as 'hybrid' and 'bronze' and how closely these relate to the heuristics that are used to detect them, which are necessarily imperfect. Specifically it is important to distinguish between the category of articles that are temporarily released by publishers from behind a paywall, and those articles that are detected by a process of identifying free copies on a publisher website without an explicit license ('bronze'). As the argument of the paper hinges on the identification and categorisation of these articles and implicitly on the motivations of publishers in releasing them it is critically important that the category of access models (promotional or emergency release) is distinguished from the categories that can be detected ('bronze').

*The authors agree completely with this perception. In the V2 of the paper we update these conclusions by analysing the licenses of each paper together with each location. In this context, we can take conclusions about whether the publisher intend to contribute to the emergency situation or not (ie. The role of PMC during this global health emergency as the main COVID-19 repository).*

Specific suggested changes to address this and related issues:

1. Under 'Data analysis' it is not immediately clear to me why the Web of Science classification is referred to. I would argue that what should be presented is the detailed implementation of exactly how the categories are assigned in this article (see Replication report below for an example of this). If the categories provided by Unpaywall are used directly this should be explained.

*We used WoS classification as it is based on the Unpaywall’s one. But you are right, it is easier to follow by just mentioning the categories provide by Unpaywall. V2 of the paper follows this last one.*

 

1. More detail on the process of data preparation would be helpful. The provision of the finalised data is very useful but details of how the Unpaywall data was collected (via the API in OpenRefine or by upload of a set of DOIs?) and exactly when (because this makes a difference to analysis, see below).

 

*We agree upon this and we detail in this sense the description of the methods and tools in V2 of the paper. V2 includes the following points:*

- *PubMed was selected as our database after a comparative study performed versus WoS. Even if it would have been easier to perform de study with WoS (as it includes an “open access” filter that PubMed does not), this presented false “closed” articles that at the same time were open in PubMed. It seems that in March, WoS was not updated enough and lacked of OA information.*
- *Unpaywall data was collected via the API in OpenRefine. PubMed data was uploaded to OpenRefine and via the API all the Unpaywall data was collected.*
- *It is important to mention that Unpaywall has notified us about an update of one of its filters during the timeframe of our study, thus affecting some of our results. This implies some license information that we are updating then the data. The publisher allowed us that update and it is included in V2 of the paper.*

 

1. Throughout the discussion there is a potential for confusion with terms like 'non-licensed Bronze'. I would use 'Bronze' throughout, perhaps repeating the point that it is by definition non-licensed. Similarly the statement '...many of these Bronze OA publications have been published in Hybrid journals...' is confusing as by the definitions used here Bronze will always be in a hybrid journal.

 

*You are right, it seems redundant. Also important to point out that in the new analysis there are 31 Bronze papers with licenses in the repositories they are uploaded, which means that there are a few within this category that are not only promotional for the publisher or pure bronze (as you said, by definition non-licensed).*

 

1. A related issue is that I would prefer to explicitly use a term like 'DOAJ Gold' to refer to articles in purely open access journals as there is significant variation across the literature in the application of this term and being explicit throughout would help.

*This is an important issue when analysing the data. You are right that we should clarify when defining each OA category. Based on Unpaywall Gold definition, “not only DOAJ indexed journals are included, but also 100% OA journals”. Unpaywall clarifies in this sense how they set the Gold OA status of an article (see: https://support.unpaywall.org/support/solutions/articles/44001792752):*

*We set the oa_status of an article to “gold” if that article is published in a fully OA journal. We have three steps to decide if a given journal is fully OA: *

1. *is in DOAJ. If not:*
2. *Is it a known fully-OA publisher? We maintain a small whitelist of publishers that we know only publish OA content (for instance, many publishers using the SciELO model). If the journal’s publisher is on this list, it’s a fully OA journal, even though it’s not in DOAJ. *
3. *Does the journal publish only OA articles? Since we index the complete output of over 70,000 journals, we’re able to check our database to see if a given journal publishes exclusively OA content. If they do, they’re a fully OA journal, even if they’re not listed in DOAJ.*

*So, Gold OA category includes DOAJ indexed journals, but also other 100% OA considered ones that UnpayWall is getting in its database. So we do not use DOAJ Gold but ‘Gold’ taking into account that “Gold” is, at the end, what UnpayWall has as Gold, following the 3 steps cited above.*

There is also some confusion in the description of Green OA. Specifically, the definition of Green adopted here is one which applies only to those articles that are not also Gold. This is standard practice, although I personally think it inadvisable, but here it leads to significant confusion. In fact, the contribution of repository access to this corpus is nearly as great as that of publishers with 43% being described as "shadowed green". I would argue for a more detailed analysis of the repositories being used in the results section.

*Totally agree, when re-describing OA categories we are going to take this into account as most of the Gold and Hybrid articles present a repository copy. In the new analysis in V2 of the paper, all these repository copies are deeply analysed.*

In policy and analysis terms this is arguably as important a contribution to access as that of publishers. I would argue for a greater analysis of this part of the corpus (see replication report for further details). The choice of PubMed Central to accept the deposit of articles with no guarantee of long-term access is a significant potential issue. This both raises questions about definitions of "green" open access and licensing that deserve a little more attention in the discussion in my view.

*Yes, together with the previous point, this is carefully addressed and discussed in the new version (V2) of the article.*

The paragraph in the discussion that commences "In line with this..." is difficult to parse. It is not clear to me that the lack of a license on the publisher site (which results in a categorisation as bronze) necessarily flows through to the licensing of the Pubmed Central version. This deserves further analysis (see below). The paragraph reads as though the assignment of only 3% to green implies that the repository copies are not guaranteed. My reading of the methodology does not agree with this. This strengthens the argument for an explicit description of the category assignment.

*We also agree upon this this point, together with last two points, The further analysis has demonstrated that most of the repository copies don’t carry licenses (well, they call it “custom licenses” as the ones stated by Elsevier or Springer Nature). This highlights the role of PMC.*

Finally I think the conclusion is probably too strong on what the analysis demonstrates vs what the concerns of the authors are. While I agree with their conclusion that it is unfortunate that the release of otherwise restricted content in the context of the pandemic has such limitations in terms of time frame and re-use this analysis cannot show the downstream effects of those restrictions, which will need to await future analysis. I think a sharper distinction between the observations made and the concerns of the authors would benefit the article.

*You are right, our strong opinions lead to strong conclusion. We have tried to “relax” them in the new version of the paper.*

**Minor issues**

Figure 1 has a number of misleading characteristics. In Figure 1a a bar chart is presented that shows both *all* articles and the oa subset but adding the two together. Figure 1c is also confusing. As noted below I don't understand why the data has been divided up the way it has. Both the conflation of the two evidence types for which Unpaywall found free articles, combined with leaving out of DOAJ as evidence source for the second pie chart seem odd and these results are not used elsewhere in the paper. I would leave 1c out and use a venn diagram for 1a and a bar chart for 1b.

*Ok, we will change the Figures 1a and 1b, and leave 1c out. The update carried out by Unpaywall reflects the vagueness of this figure, and the new analysis has been done by looking at each evidence (up to 4 locations) of each article. Instead, we can include a Venn diagram overlapping each OA category with the ones with a repository copy.*

Figure 2 and related text. The license category of 'cc' is presumably cc0.

*Yes. It is CC0, It is update in the v2 or the paper.*

I find Figure 4d confusing. Would it not be better to show some quantitative parameter for each of the publishers rather than the -OPEN and +OPEN? Perhaps open licences as a proportion of all articles or something similar?

*We agree. We have clarified the representation in Figure 4d.*

In analysing past outbreaks the issue of increases in repository-mediated (green) OA over time should be explicitly mentioned. This might particularly be included in a comparison of those repositories that are contributing to access. This does not directly affect the conclusions of these sections as the proportion of green is not otherwise interpreted but the potential for confusion means this should be at least mentioned with a statement saying that it is not therefore possible to directly compare the levels of green open access across these outbreaks.

*Totally agree.*

Issues Identified

*Minor issues*

1. The dataset has 5621 rows of data, not 5611 as specified in the paper. Is this to do with blank entries or entries without DOIs? *8 belong 2019 y 2 contain JSON error, so 10 were excluded.*
2. There are 4989 oa articles by my analysis, not 4986 as specified in the paper. Comparison to the provided data provides 4991 oa articles, and the difference is explained by the two entries for which the JSON does not parse. *After excluding the previous 10, 4986 is the final number.*
3. Not immediately clear why for Fig 1c the two categories of free article have been combined?
4. Why in Figure 1c are the oa types reported only for those articles where the evidence type is either free article or free pdf? Why are the DOAJ evidence examples not included?

*In order to avoid confusion fig 1c is be taken out in V2 of the paper.*

1. Figure 2. Slight variation in the percentages calculated from the dataset. *You have performed the analysis for the hole publications, not just for the OA ones. We calculate the percentages only of the OA collection.*
2. There are slight issues in 4b and 4c with license assignment. *If our Gold definition is used, the numbers should be correct. Even so, these numbers change in V2 of the paper when we consider the figures updated by UnpayWall.*
3. As noted in general comments I would drop Figure 4d as it is confusing and it is not clear to me that it is supported by the data where Wiley does not appear to have many more open licenses than Elsevier. *You are right. We update this figure in V2.*

*Major Issues*

1. The numbers in the paper do not seem to correspond directly to those in the dataset provided *The data we report in the paper correspond with the filtered data, and they are coherent with the chosen criteria.*
2. It appears the article does not use DOAJ as the criterion for gold but the is_oa_journal field from unpaywall. This explains the variance between my analysis and that in the article for "gold" as defined here (16% vs 19% using the data provided, vs 21.5% given in Figure 1) *Explained in previous points: Unpaywall includes 100% OA journals, DOAJ indexed or not. This issue is clearer stated in V2 of the paper.*
3. In Figure 5-7 I think there may be an error in the counting of OA articles, counting all those articles for which there is an 'is oa' entry and not only those where it is set to True. *For the analysis is needed to exclude the articles not analysed by Unpaywall (and thus, have an empty OADOI field). If you do this, the numbers are ok.*

*Thank you so much for your great review.*
